# A Canal of Nuck Cyst Presented as a Femoral Hernia: A Rare Case Report With Diagnostic Dilemma

**DOI:** 10.7759/cureus.51908

**Published:** 2024-01-08

**Authors:** Mohammed I Elsayed, Rashid Ibrahim, Amulya Khatri, Madan Palliyil

**Affiliations:** 1 General Surgery, Stepping Hill Hospital, Manchester, GBR

**Keywords:** complex cyst, female gender, endometriosis, femoral hernia, the canal of nuck

## Abstract

A cyst of the canal of Nuck is an uncommon disorder in females. It results from the failure of obliteration of the peritoneal fold that runs along the round ligament. This case report details a unique and rare presentation of a 38-year-old female who presented with a right groin swelling. Although her preoperative images showed only the right canal of the Nuck cyst, the intraoperative diagnosis was established as a femoral hernia containing a canal of the Nuck cyst. She underwent an elective cyst excision with repair of the femoral hernia. She had an uneventful post-operative recovery. A femoral hernia that contains a cyst of the canal of Nuck is a rare manifestation of this uncommon condition. The most effective treatment options are surgical cyst excision and repair of the femoral hernia.

## Introduction

A cyst of the canal of Nuck is a rare disorder in female patients. Embryologically, it results from the failure of obliteration of the peritoneal fold along the round ligament that attaches the uterus to the labia majora. Ordinarily, this peritoneal fold obliterates during the first year of life; failure of obliteration will lead to an indirect inguinal hernia or a hydrocele, which is referred to as a canal of Nuck cyst [1]. This condition is embryologically similar to patent processus vaginalis in males. The canal of Nuck cyst is very rare, and there is no exact incidence in the literature. Historically, this condition was first identified by Anton Nuck, a Dutch anatomist [2]. Subsequently, this rare condition was reported in several case reports [1,3,4]. The cyst is diagnosed clinically and confirmed radiologically via ultrasound (US), computed tomography (CT), and magnetic resonance imaging (MRI) [4-6]. Most of the canal of Nuck cysts presented to the surgical department as groin hernias, mainly indirect inguinal hernias [3]. The procedure of choice for treatment is to excise the cyst and narrow the deep inguinal ring [7]. This case report demonstrated a rare presentation of the canal of the Nuck cyst as a femoral hernia.

## Case presentation

**Figure 1:**
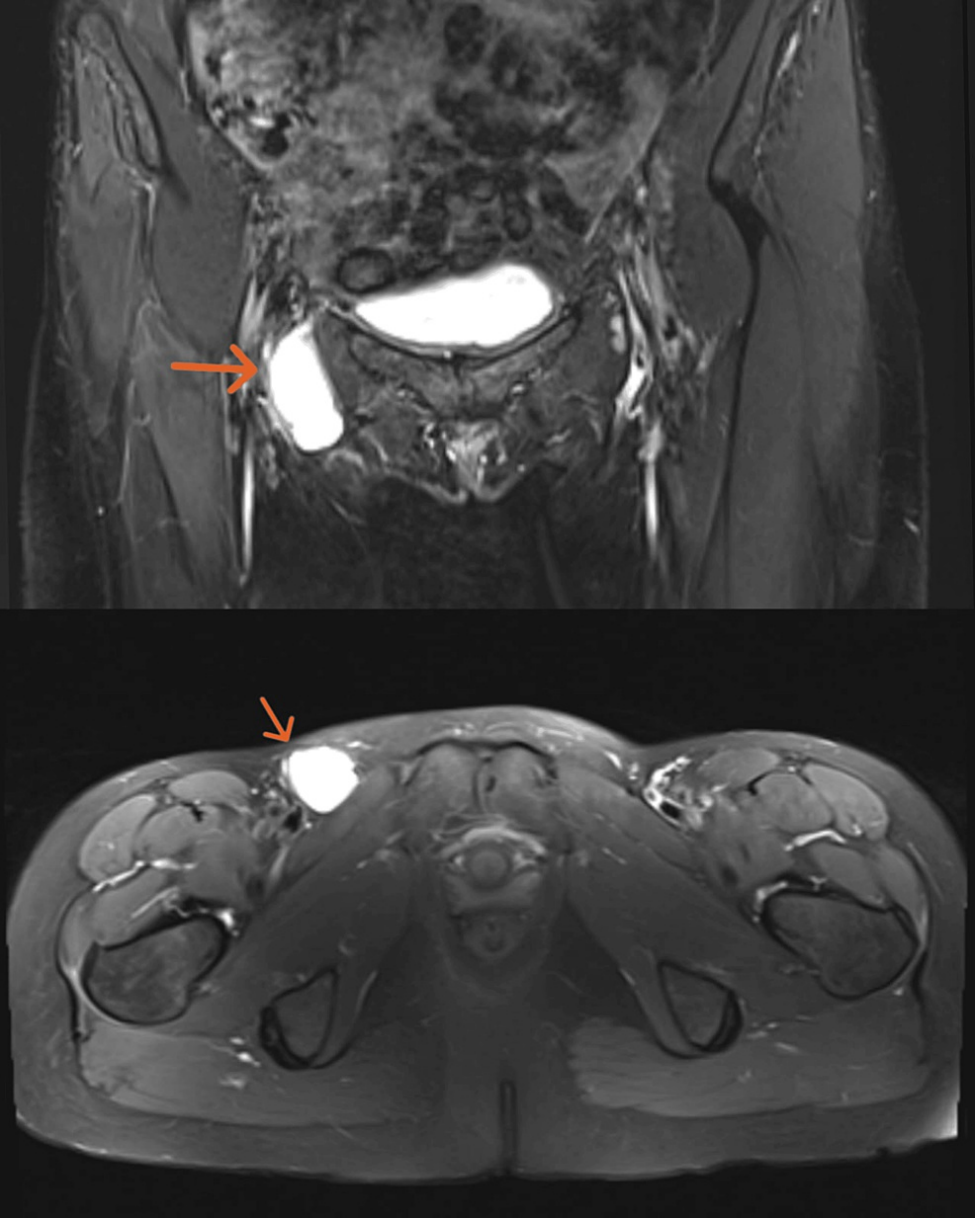
MRI pelvis showing complex cyst at the region of the canal of Nuck (orange arrow) with no evidence of femoral or inguinal hernia

**Figure 2:**
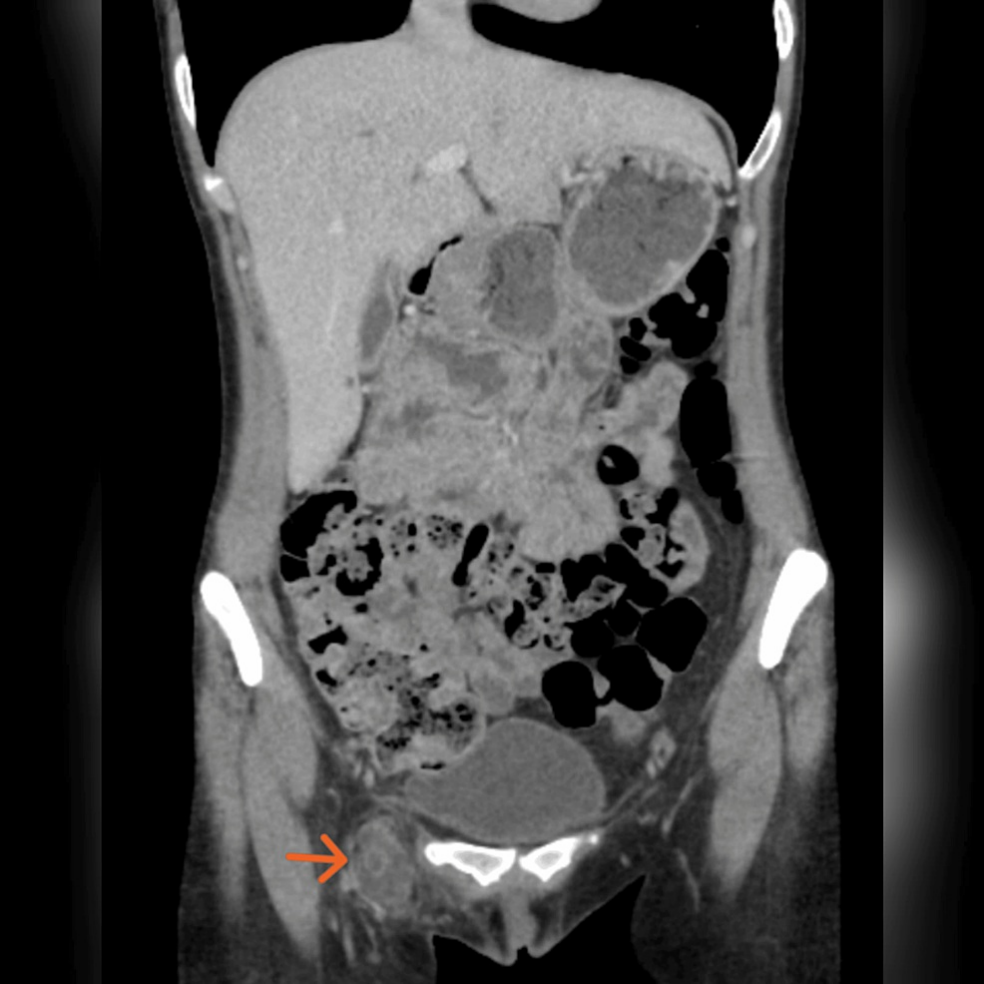
CT abdomen and pelvis showing a cystic lesion in the right femoral region (orange arrow) with no femoral or inguinal hernia

A 38-year-old woman presented to the hospital with an 18-month history of right-sided groin swelling. She stated that the swelling was gradually increasing in size. Recently, it has become more prominent and very tense, causing ongoing discomfort in the area. She had no history of heavy weight lifting or vigorous exercises but had undergone a laparoscopic left oophorectomy for endometriosis four years ago. On examination, she had cystic swelling in the right groin area, measuring about 4x3 cm. It was tense, tender, and irreducible with negative cough impulse. The patient had a groin ultrasound scan, which ruled out inguinal and femoral hernias. The scan showed cystic and soft tissue swelling in the right femoral region. Therefore, an MRI scan of the pelvis was requested, which showed a complex cystic lesion containing fat within the region of the canal of Nuck; it measures around 40mm in diameter, with no evidence of femoral or inguinal hernias (Figure 1). She had a CT scan of the abdomen and pelvis as part of her surveillance for endometriosis, which revealed a 4.6x2.6 cm cystic swelling in the femoral region. There was no connection with the femoral vessels, and no evidence of hernias was observed (Figure 2).

**Figure 3:**
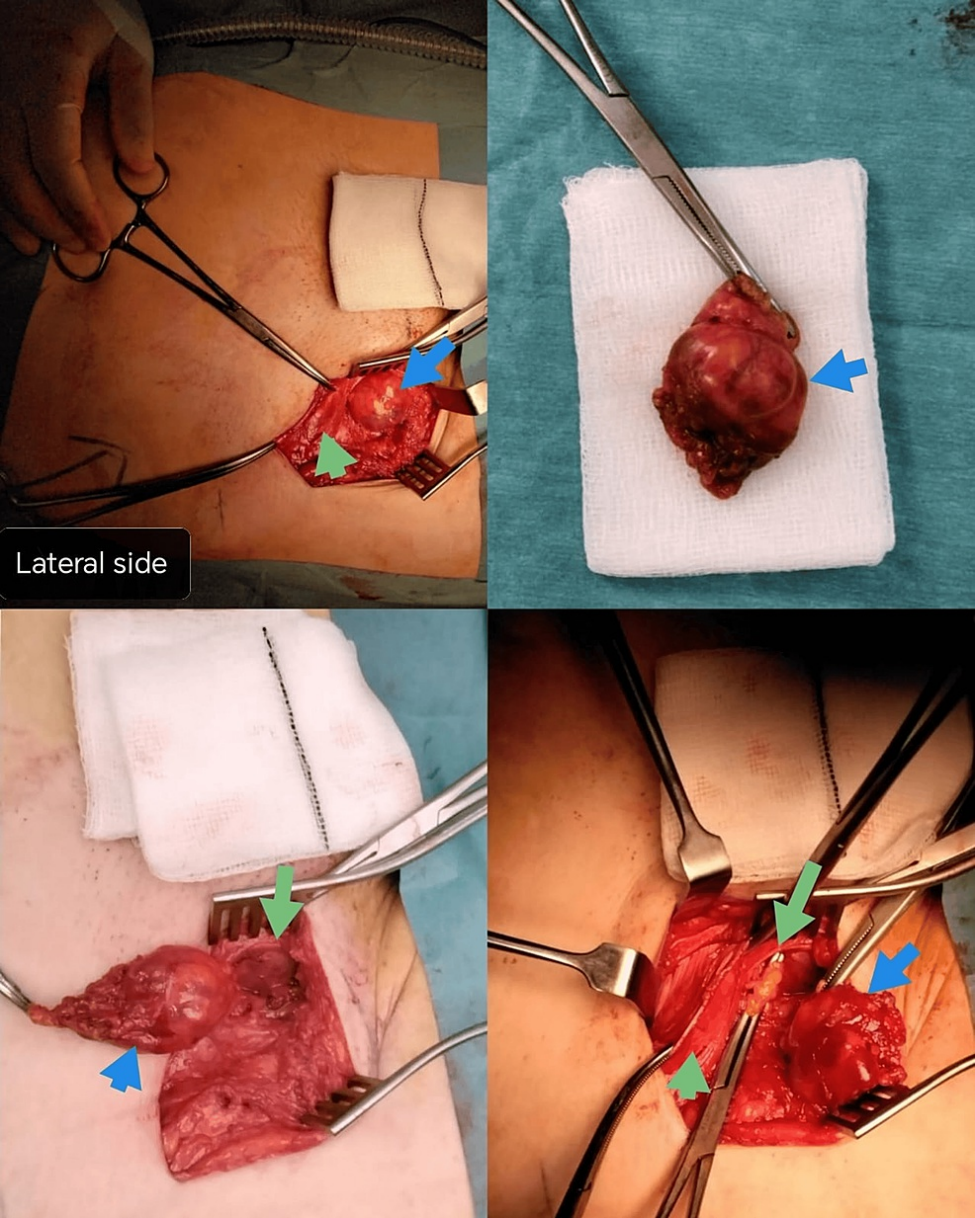
Intraoperative findings of the canal of Nuck cyst (blue arrow) passing below the inguinal ligament (green arrow) toward the femoral canal

A general surgery consultant diagnosed the patient with a cyst of the canal of Nuck, and she underwent an elective right groin exploration at a tertiary hospital. During the operation, a multi-loculated irregular-shaped cystic swelling was found passing below the inguinal ligament toward the femoral canal, medial to the femoral vessels (Figure 3). The cyst was separated from the surroundings by dissecting the adhesions while protecting the femoral vessels, and was successfully excised and sent for histology. The femoral canal was repaired by approximating the inguinal ligament anteriorly to the pectineus ligament posteriorly using non-absorbable interrupted sutures. The patient was discharged home on the same day with routine post-hernia repair instructions, such as avoiding heavy lifting and vigorous exercises for at least six weeks. She had an uneventful post-operative recovery, and the post-operative histology report is still pending. A follow-up after two months showed no evidence of recurrence.

## Discussion

The clinical presentations of the canal of Nuck cyst are classified into three types. Firstly, a hydrocele presents when there is a direct connection to the peritoneal cavity. Secondly, a peritoneal fold passes through the inguinal canal to the labia majora without connection to the peritoneal cavity. Thirdly, a combination of the previous two presentations, when there is a direct connection to the peritoneal cavity but with a very tight, deep inguinal ring that resists spontaneous sac reduction [8]. From the patient's history, the swelling was stable for a long time. It recently became more prominent and tense, giving the impression of direct communication to the peritoneal cavity with a tight, deep inguinal ring. These clinical findings were supported by the preoperative radiological scans that confirmed the presence of the canal of Nuck cyst and ruled out any inguinal or femoral hernias. In this case report, we presented a 38-year-old female who was diagnosed intraoperatively with a right femoral hernia containing a canal of Nuck cyst. This presentation is considered the rarest form of this condition, with only one other case being reported in the literature with a similar diagnosis by Baig et al. in a 70-year-old female [9]. 

The canal of Nuck cyst is most commonly associated with an inguinal hernia, particularly an indirect inguinal hernia, with an incidence of approximately 40%, according to studies [10-12]. The most common clinical presentation of this condition is painless, irreducible swelling in the groin area, which is usually found on the right side, as reported by Prodromidou et al. in their systematic review, with an incidence of 81.3% in comparison to the left groin region [13]. Our case also presented with a painless, irreducible swelling in the right groin area. Diagnosing the canal of Nuck cyst could be quite challenging due to the rarity of the disease and the discrepancy between preoperative images and intraoperative findings. Preoperative imaging investigations may fail to detect the presence of a femoral hernia. Ultrasound imaging is unreliable for this condition, as stated by Baig et al. [9]. The CT scan and MRI may also fail to detect a femoral hernia, as in our case. Therefore, groin exploration is essential to avoid missing a coexisting groin hernia, even if the preoperative images ruled it out. It is interesting to note that our patient had a history of endometriosis, which was treated by the gynecology team a few years ago. In the case reported by Stickel et al., it was mentioned that one of the rare differential diagnoses of this condition could be endometriosis of the round ligament [14]. However, all of the preoperative workup for our case ruled out the possibility of recurrent endometriosis. We are still awaiting the histology report, and the author emphasized the importance of histopathology, even if the intraoperative findings seem to be a simple cyst. In this case report, we used an open low inguinal approach, which is the most popular method used in the majority of the previous case reports. Out of the 16 case reports mentioned in the systematic reviews of Prodromidou et al., 13 were performed using the open approach [13]. The author believes that the open approach is technically easier than the laparoscopic approach for the excision of the cyst and repair of the coexisting hernia, especially in surgeons with limited laparoscopic experience. As far as we know, there was only one published case report of a femoral hernia containing a canal of Nuck cyst, which was treated by cyst excision and repair of the femoral hernia [9].

## Conclusions

In conclusion, while cysts of the canal of Nuck are rare surgical conditions, they should be contemplated in the differential diagnoses of any groin swellings in female patients. A femoral hernia containing a canal of Nuck cyst is a rare presentation of these cysts. Preoperative radiological investigations are crucial to confirm the diagnosis of the cyst. However, during surgery, the limitations of these investigations in delineating the coexisting femoral hernia should be considered. The best available treatment options for this rare condition are groin exploration with excision of the cyst and femoral hernia repair.
